# P-1739. Elevated Red Blood Cells in Bronchoalveolar Lavage Fluid are Associated with False-Positive Aspergillus Galactomannan Results

**DOI:** 10.1093/ofid/ofaf695.1910

**Published:** 2026-01-11

**Authors:** Mabel Jimenez, Denise McCulloch, David Fredricks, Elizabeth M Krantz, Steven A Pergam

**Affiliations:** Fred Hutch Cancer Center, Infectious Disease Division, Seattle, WA, Seattle, Washington; Fred Hutchinson Cancer Center, Seattle, WA; Fred Hutchinson Cancer Research Center; University of Washington, Seattle, WA; Fred Hutch Cancer Center, Seattle, Washington; Fred Hutchinson Cancer Center; University of Washington, Seattle, WA

## Abstract

**Background:**

The Aspergillus galactomannan (GM) assay in bronchoalveolar lavage (BAL) fluid is used to diagnose invasive pulmonary aspergillosis, but specificity is variable. Lack of specificity is particularly important for immunocompromised patients, including those with hematologic malignancies, where a false positive can lead to misdiagnosis, delays in treatment and unnecessary antifungal therapy. Based on our clinical observations, we hypothesized that elevated red blood cell (RBC) counts in BAL fluid are associated with false-positive GM results.

**Table 1.**
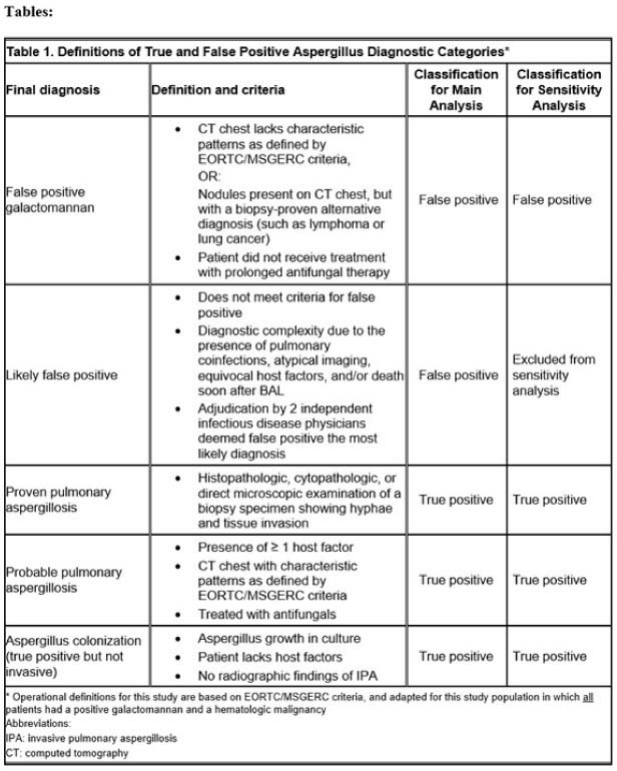
Definitions of True and False Positive Aspergillus Diagnostic Categories.

**Table 2.**
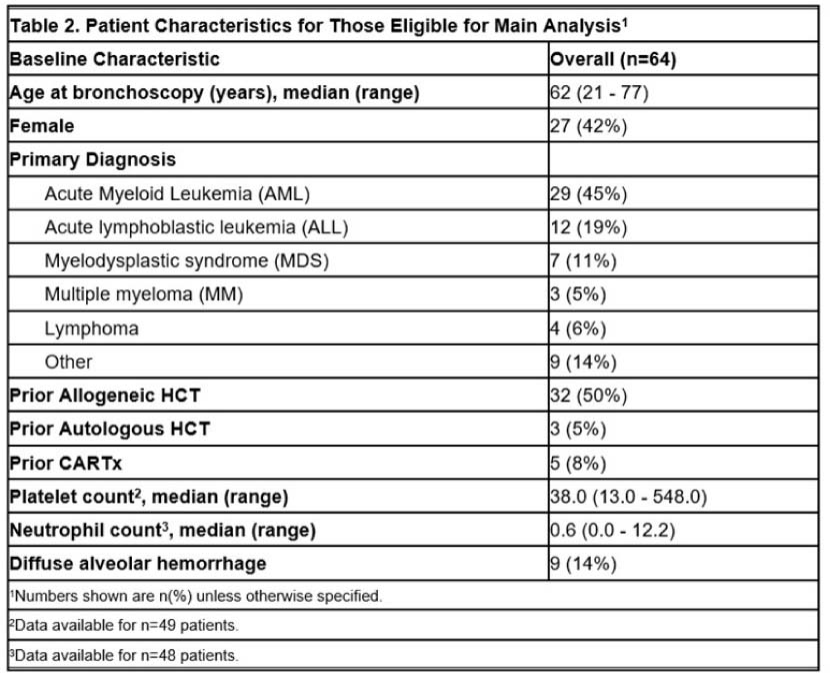
Patient Characteristics for those eligible for main analysis.

**Methods:**

We conducted a retrospective cross-sectional study of adults with hematologic malignancies, including those undergoing hematopoietic cell transplant (HCT), or CAR-T cell therapy (CARTx) who underwent bronchoalveolar lavage (BAL) at Fred Hutchinson Cancer Center from July 2021 to July 2024 and had a positive BAL GM. Two clinicians adjudicated final diagnoses into five categories to identify false positives (Table 1). RBC counts were grouped into tertiles. Poisson regression with robust standard errors was used to estimate adjusted relative risks (aRR) and 95% confidence intervals (CI) to test for an association between RBC count in BAL fluid and false versus true positive Aspergillus diagnosis, controlling for HCT or CARTx history.

**Figure:**
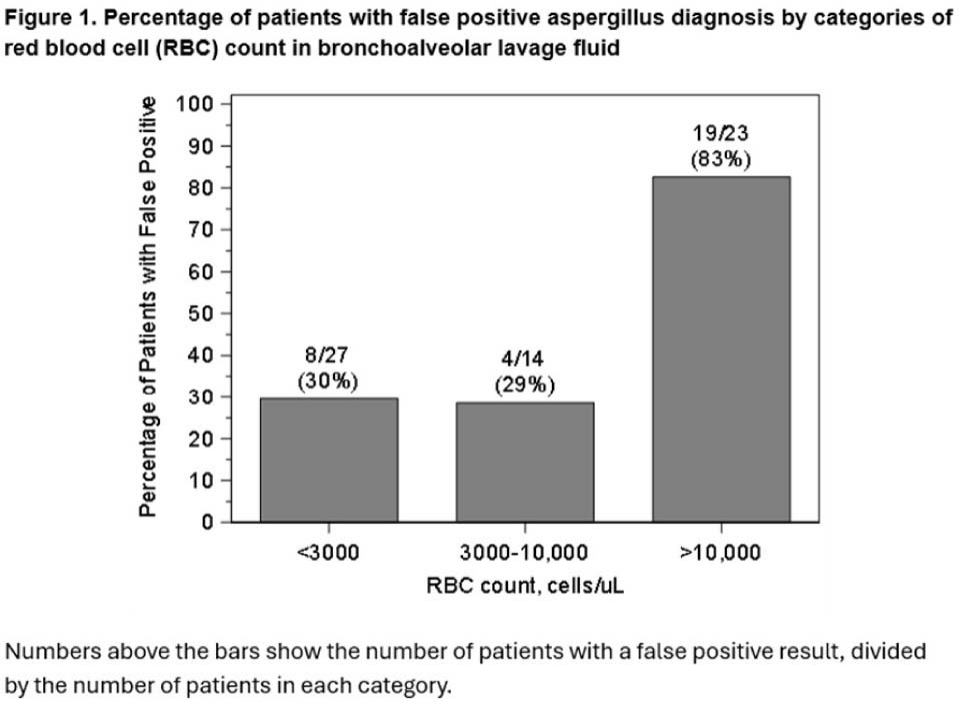
Percentage of patients with false positive aspergillus diagnosis by categories of RBC count in bronchoalveolar lavage fluid.

**Figure:**
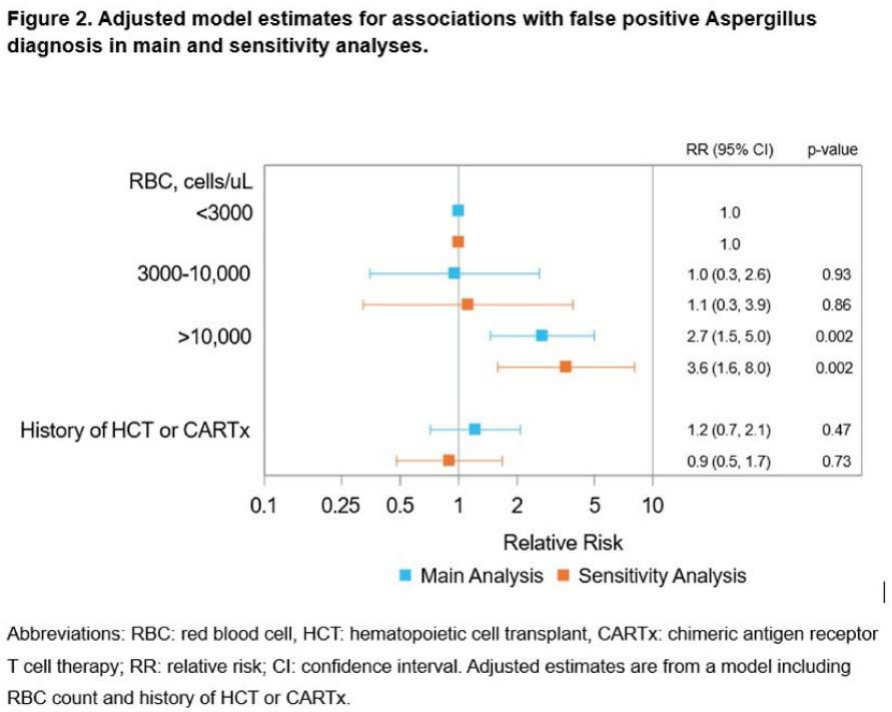
Adjusted model estimates for associations with false positives Aspergillus diagnosis in main and sensitivy analyses.

**Results:**

Patient demographics are in Table 2. Among 64 patients (59% with prior HCT or CARTx), false positives occurred in 31/64 (48%) overall, 8/27 (30%) with < 3,000 RBCs/µL, 4/14 (29%) with 3,000–10,000 RBCs/µL, and 19/23 (83%) with >10,000 RBCs/µL (Figure 1). Adjusted models showed a significant association for >10,000 vs < 3,000 RBCs/µL (aRR: 2.7; 95% CI: 1.5–5.0; p = 0.002). Sensitivity analysis excluding "likely" false positives yielded similar results (Figure 2).

**Conclusion:**

BAL RBC counts >10,000 cells/µL were associated with increased risk of false-positive GM results. Excess blood may interfere with the GM sandwich immunoassay. GM false positives are a known limitation of the assay, but clinicians should exercise caution when interpreting GM results from bloody BAL samples in patients with hematologic malignancies.

**Disclosures:**

Denise McCulloch, MD, MPH, Pfizer: Grant/Research Support David Fredricks, MD, BD: Royalty|Seres Therapeutics: Advisor/Consultant Steven A. Pergam, MD, MPH, F2G: Site PI for clinical trial|Global Life Technologies, Inc.: Grant/Research Support|Mundipharma: Site PI for clinical trial|Symbio: Site PI for clinical trial

